# Sequestration of imaging studies in MIDRC: stratified sampling to balance demographic characteristics of patients in a multi-institutional data commons

**DOI:** 10.1117/1.JMI.10.6.064501

**Published:** 2023-11-16

**Authors:** Natalie Baughan, Heather M. Whitney, Karen Drukker, Berkman Sahiner, Tingting Hu, Grace Hyun Kim, Michael McNitt-Gray, Kyle J. Myers, Maryellen L. Giger

**Affiliations:** a University of Chicago, Department of Radiology, Chicago, Illinois, United States; b US Food and Drug Administration, Bethesda, Maryland, United States; c University of California, Los Angeles, Los Angeles, California, United States; d Puente Solutions, Phoenix, Arizona, United States

**Keywords:** machine learning, stratified sampling, sequestration, COVID-19, image database, algorithm performance, Medical Imaging and Data Resource Center

## Abstract

**Purpose:**

The Medical Imaging and Data Resource Center (MIDRC) is a multi-institutional effort to accelerate medical imaging machine intelligence research and create a publicly available image repository/commons as well as a sequestered commons for performance evaluation and benchmarking of algorithms. After de-identification, approximately 80% of the medical images and associated metadata become part of the open commons and 20% are sequestered from the open commons. To ensure that both commons are representative of the population available, we introduced a stratified sampling method to balance the demographic characteristics across the two datasets.

**Approach:**

Our method uses multi-dimensional stratified sampling where several demographic variables of interest are sequentially used to separate the data into individual strata, each representing a unique combination of variables. Within each resulting stratum, patients are assigned to the open or sequestered commons. This algorithm was used on an example dataset containing 5000 patients using the variables of race, age, sex at birth, ethnicity, COVID-19 status, and image modality and compared resulting demographic distributions to naïve random sampling of the dataset over 2000 independent trials.

**Results:**

Resulting prevalence of each demographic variable matched the prevalence from the input dataset within one standard deviation. Mann–Whitney U test results supported the hypothesis that sequestration by stratified sampling provided more balanced subsets than naïve randomization, except for demographic subcategories with very low prevalence.

**Conclusions:**

The developed multi-dimensional stratified sampling algorithm can partition a large dataset while maintaining balance across several variables, superior to the balance achieved from naïve randomization.

## 1 Introduction

Since early 2020, the COVID-19 pandemic has presented an urgent and critical public health crisis. This crisis produced several needs for essential biomedical research and development to address, including early detection and differential diagnosis of COVID-19, prognosis and assessment of response to treatment, and monitoring of the post-COVID patient. All these needs created an important role for artificial intelligence (AI) in medical imaging of the COVID patient and stimulated a rapid research effort in AI model development for COVID-19 applications.^1^^–^^3^ Many studies have found promising performance of AI models for various applications. However, potential impact of algorithm bias and lack of clinical utility have been noted as major shortcomings in AI developed for COVID-19 medical imaging, particularly algorithms developed early in the pandemic.^4^^–^^6^ As a response to this urgent public health need, the Medical Imaging and Data Resource Center (MIDRC^7^) was established in August, 2020, to accelerate medical imaging machine intelligence research for COVID-19.

MIDRC is a multi-institutional research collaboration between the American College of Radiology (ACR^®^), the Radiological Society of North America (RSNA), and the American Association of Physicists in Medicine (AAPM), created to address critical gaps in resources and technology for AI in medical imaging. Through the work of five technology development projects and 12 collaborating research projects, MIDRC is providing processes for data intake, de-identification, quality assessment, and distributed public access in addition to organizing research challenges and curated datasets to support high-quality research methods. The aim of MIDRC is to accelerate machine intelligence research for COVID-19 and eventually for other diseases that utilize medical imaging in, e.g., detection, diagnosis, or prognosis. One primary component of MIDRC is the development of a publicly available image repository,^7^ as well as a sequestered database for performance evaluation and benchmarking of algorithms.

While the majority of the de-identified data (both images and metadata) submitted to MIDRC are open, i.e., accessible to the public, approximately 20% of the data are being sequestered from public use for the purpose of machine intelligence algorithm evaluation. These sequestered data will act as a large base from which task-based samples, or “test-sets,” can be drawn to provide an estimate of an algorithm’s performance or generalization ability, without ever releasing the data publicly or giving users direct access to the cases used for testing, thus maintaining the integrity of the test set.^8^

To assure both datasets are similarly representative of the population, we developed a methodology to balance demographic characteristics, or variables, across the sequestered and public data. This process is implemented for incoming batches of data on an ongoing basis. While MIDRC aims to provide a platform with a wide array of diverse data representative of the population, gaps and biases may inadvertently arise. MIDRC aims to recognize and address these biases throughout the research workflow through multiple bias-reduction strategies and resources for users to further reduce potential bias.^9^ Providing a public resource of curated COVID-19 data and open-source algorithms for data analysis and cohort building will aid in bias mitigation by increasing availability of multi-institutional data and standardized data labels across many demographic variables.^10^ Further, purposeful selection of patients for the sequestered database will be a useful tool for the future assessment of the bias of the evolving database itself, as well as the algorithms developed based on the database.

Potential bias and lack of generalizability in AI algorithms have been key shortcomings of the clinical utility of AI, and multiple studies have found demographics to have a profound impact on the performance of medical image classifiers.^10^^–^^15^ Biases can also be hidden in data structures that are not explicitly defined or monitored, leading to unintended bias in resulting model performance.^16^^–^^18^ These studies highlight the key importance of mindful balancing of demographic distributions within datasets used for machine intelligence applications.^14^

Balancing multiple variables among subgroups is a widely studied topic in the field of clinical trial development.^19^^,^^20^ Shifting demographic profiles of the study population have also been investigated in the context of clinical trials.^21^ However, similar approaches have rarely been applied in the field of machine intelligence due to the use of typical train-test splitting in datasets that are often small or moderate in size. In both clinical trials and imaging data, there are similar patient variables, but imaging contributes additional complexity regarding imaging machine type, protocol, etc., as the data collection process is more varied. Here, we apply a developed method of multi-dimensional stratified sampling to separate incoming MIDRC data into open and sequestered commons and evaluate the performance of the sampling method in terms of similarities between the two commons. Our method differs from existing approaches in that it incorporates several demographic variables and is used on an ongoing basis to continually build two demographically matched data commons, at scale. In addition, the methodology developed for this work takes the standard concept of stratification and expands upon it in a script that is shared as a public resource on the MIDRC GitHub website.

The goal of the developed sequestration algorithm is to generate two commons with demographically similar characteristics. Developers can then sample and train on any portion of the publicly available data, and algorithm performance will be assessed with portions of the sequestered commons relevant to the user’s task. Our goal is to create a balance between the open data that developers will use for training algorithms and the sequestered data that will provide a balanced test set. The purpose of this work is to describe this process and provide a brief evaluation of the current state of sequestration of imaging studies within MIDRC.

## 2 Methods

Prior to sequestration, de-identified clinical data of patients are submitted to MIDRC through data input portals hosted by ACR and RSNA.^7^ Quality of submitted medical imaging studies is assessed, and the associated metadata are harmonized for representation within the MIDRC data model at Ref. ^7^. Subsets of the incoming data undergo separation and are designated as “open” or “sequestered” on an ongoing basis in batches created at regular submission time intervals.

**Fig. 1:**
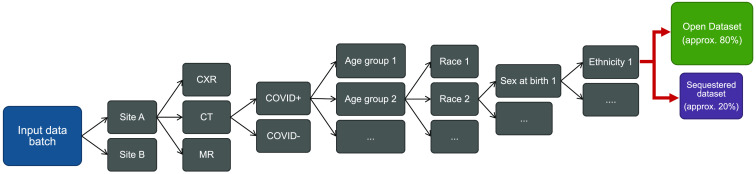
Diagram of demographic factors used to stratify data for separation into sequestered and open databases. The input data batch is sequentially split into all possible variations of each category until an individual stratum, containing a unique combination of variables, is achieved. This individual stratum is then randomly separated into the open and sequestered commons with proportions of approximately 80:20.

To sequester approximately 20% of an incoming data batch, first, de-identified patient IDs are compared across all previously processed batches. If a patient ID already exists in either the public or sequestered commons, the incoming data for this patient are placed in the relevant commons. This process ensures data are placed into the open or sequestered commons at the patient level, and all images from longitudinal studies of a given patient are contained in only one of the two commons. Following this longitudinal data check, the data of remaining patients in the intake batch are sequentially separated into multiple strata based upon the anonymized clinical site ID, image modality, COVID status (whether a patient ever tested positive for COVID), and reported patient race, age, sex at birth, and ethnicity. These variables were selected from the standard variables included in the Ref. 7 patient metadata by the authors after consideration of the potential impact of each variable on future developed algorithms and importance of balanced representation of that variable between the two commons. The number of included variables is a compromise between balanced representation across many variables but few enough variables that stratification does not creates a majority of bins with such low prevalence that they will not be sufficiently balanced. The selected variables are not a comprehensive list, but represent common variables considered in ML algorithm subgroup analyses and have been shown in the literature to have potential impact on the performance of developed classifiers.^11^^–^^14^ A diagram of the sequestration process is shown in Fig. 1. Within each resulting bin or strata, i.e., a group of patients with a particular combination of characteristics, the patients are randomly assigned to the open dataset or the sequestered dataset with proportions of approximately 80% and 20%, respectively. Thus, for n variables of interest, the balance of the n-dimensional distribution of variable combinations can be controlled.

Since, for any given patient, imaging studies from multiple modalities might be available, such as computed tomographs (CT) or radiographs, the sequestering of patient data by modality was accomplished by first identifying the most prevalent image modalities in an intake data batch. If a patient entry contained more than one modality, the modality most prevalent in the intake data batch was established to be that patient’s “primary” modality, and images from any less prevalent modality were assigned along with the most prevalent modality. Modalities present in this analysis included computed radiograph (CR), CT, digital radiography (DX), and magnetic resonance (MR) images. Patient age data were grouped into categories matching the age group categories provided by the Center for Disease Control COVID-19 database.^22^ Patient sex at birth, race, and ethnicity were grouped in agreement with the categories defined by the NIH.^23^^,^^24^

To demonstrate the sequestration process in simulation, 5000 patients were randomly selected from the public data commons^7^ to serve as an example of an input dataset. This number of patients approximately models a typical data submission from a single clinical site to MIDRC from 2021 to 2022. As such, the example input dataset was not separated by contributing clinical site. It is important to note that users will not be training or testing on any single batch of data, rather all cumulative data batches (after being sampled using the developed sequestering method) will be available to users from the public data commons. The developed sequestration algorithm was applied to the remaining demographic variables (age, race, sex at birth, ethnicity, COVID-19 status, and image modality) to achieve a similar distribution of variables between the input dataset and the two subsets, i.e., the open and the sequestered data commons. The variables of age, race, sex at birth, ethnicity, COVID-19 status, and image modality contained 9, 7, 4, 3, 3, and 4 categories, as listed in Table 1, respectively, resulting in a total of 9072 strata. As such, strata used in separating data are multiplicative, and the overall number of categories and strata are relatively large. As more strata are included, it is important to have enough data to adequately fill those strata to generate a balanced separation. There is no known rule on how many strata are too many, but the number should be considered with the size of incoming data and what demographic variables are of key importance to research questions.^20^

**Table 1:** Distribution of all balanced variables in the input, open (∼80%), and sequestered (∼20%) datasets following splitting via stratified sampling. Prevalence values are written as the mean percent (standard deviation) over 2000 independent trials. The label of “not reported” was added to variables with blank entries.

Demographic subcategory	Input dataset count	Input dataset prevalence	Open subset prevalence	Sequestered subset prevalence
**Age group**				
[0, 18)	74	1.5%	1.5% (0.1%)	1.4% (0.3%)
[18, 30)	393	7.9%	7.9% (0.1%)	7.9% (0.3%)
[30, 40)	529	10.6%	10.6% (0.1%)	10.3% (0.4%)
[40, 50)	687	13.7%	13.7% (0.1%)	13.9% (0.4%)
[50, 65)	1434	28.7%	28.6% (0.1%)	29.1% (0.4%)
[65, 75)	909	18.2%	18.2% (0.1%)	18.1% (0.4%)
[75, 85)	597	11.9%	11.9% (0.1%)	11.9% (0.3%)
[85, 140)	284	5.7%	5.7% (0.1%)	5.5% (0.3%)
Not reported	93	1.9%	1.8% (0.1%)	1.9% (0.2%)
**Race**				
American Indian or Alaska Native	17	0.3%	0.3% (0.0%)	0.3% (0.2%)
Asian	294	5.9%	5.9% (0.1%)	5.9% (0.4%)
Black or African American	1386	27.7%	27.8% (0.1%)	27.6% (0.4%)
Native Hawaiian or other Pacific Islander	15	0.3%	0.3% (0.0%)	0.3% (0.2%)
White	2568	51.4%	51.2% (0.1%)	51.8% (0.6%)
Not reported	554	11.1%	11.1% (0.1%)	10.8% (0.5%)
Other	166	3.3%	3.3% (0.1%)	3.3% (0.4%)
**Sex at birth**				
Female	2533	50.7%	50.6% (0.1%)	50.8% (0.5%)
Male	2464	49.3%	49.3% (0.1%)	49.1% (0.5%)
Other	0	0.0%	0.0% (0.0%)	0.0% (0.0%)
Not reported	3	0.1%	0.1% (0.0%)	0.1% (0.1%)
**Ethnicity**				
Hispanic or Latino	499	10.0%	10.0% (0.1%)	9.7% (0.6%)
Not Hispanic or Latino	4443	88.9%	88.8% (0.2%)	89.2% (0.6%)
Not reported	58	1.2%	1.2% (0.1%)	1.2% (0.3%)
**COVID-19 status**				
No	2602	52.0%	52.0% (0.1%)	52.2% (0.5%)
Not reported	1	0.0%	0.0% (0.0%)	0.0% (0.0%)
Yes	2397	47.9%	48.0% (0.1%)	47.8% (0.5%)
**Image modality**				
CR	2049	41.0%	40.9% (0.2%)	41.3% (0.8%)
CT	910	18.2%	18.3% (0.2%)	18.0% (0.8%)
DX	2596	51.9%	52.0% (0.1%)	51.9% (0.5%)
MR	27	0.5%	0.5% (0.0%)	0.5% (0.2%)

To summarize the performance of our developed sequestration algorithm, we applied the algorithm to the input dataset for 2000 independent trials. In each independent trial, a different random seed was used to initiate the splitting. The mean and standard deviation of each demographic category’s prevalence was calculated over all independent trials and compared to the prevalence in the input dataset and between the two subsets.

To compare the performance of the sequestration algorithm to “naïve” separation of the dataset with an overall 80:20 random drawing, where the assignment to open or sequestered dataset is made randomly without considering demographic variables, we sought to evaluate resulting balance in the two subsets. In this paper, “more balanced” is intended to mean that the relative distributions of demographic categories are more closely matched between resulting open and sequestered subsets. For example, a balanced sample would result in both the open and sequestered subsets having a nearly 50:50 split of male and female patients, matching the initial dataset. Conversely, an unbalanced sample may have 60% male patients (and 40% female patients) in the open subset and 40% male patients (and 60% female patients) in the sequestered subset. We applied the naïve algorithm to the input dataset for 2000 independent trials, reviewed the resulting distribution of cases across demographic categories through visual inspection, and evaluated demographic balance quantitatively by comparing the median of each distribution using the one-tailed Mann–Whitney U test.^25^ The resulting distributions were first compared by creating histograms of the scaled difference from expectation in each category, calculated according to Eq. (1):

$$\text{Scaled difference from expectation}\;=\;\frac{\left|(f)N_{T}-N_{\text{Open}}\right|}{(f)N_{T}}.$$ (1)

Here, f represents the fraction placed in the open commons; this value is a scaling factor to scale the total number of patients in a given category to the proportion placed in the open commons. In this work, it is equal to 0.8. $N_{T}$ represents the number of patients in a given category in total from the input dataset; this is the incoming number of patients, to be split, in one category. If, within the initial 5000 total patients there were 500 patients with reported race as Asian, $N_{T}$ would be equal to 500 for the race category of Asian. This is the unscaled expected counts. Similarly, $N_{\text{Open}}$ represents the number of patients in a given category in the open dataset, which would be expected to be 80% of $N_{T}$ in size if the dataset split was exact; this is the resulting number of patients, after being split, in one category. If, within the resulting open subset there were 390 patients with reported race as Asian, $N_{\text{Open}}$ would be equal to 390 for the race category of Asian. This is the observed count. The value of this metric from all independent trials was plotted in histograms for a given bin within a category, e.g., in the race categories, all Asian patients. Distributions from our developed stratified sampling algorithm and from naïve random sampling were compared using the one-tailed Mann–Whitney U test. The Mann–Whitney U test is a nonparametric alternative to the t-test to compare central measures of two distributions, which can be employed without any assumption about the shape of the distribution. In this analysis, we are using a one-tailed Mann–Whitney U test with the alternative hypothesis indicating the median of the distribution of the scaled difference from expectation [Eq. (1)] from stratified sampling was less than that for the naïve random sampling. P-values <0.05 were considered significant. Results were adjusted for multiple comparisons using the Holm–Bonferroni multiple comparisons correction.^26^ In this correction, the calculated p-value must be less than the chosen significance threshold divided by the rank of said p-value, after ordering from least to greatest where the smallest p-value would have a rank of the total number of comparisons. In this analysis, 29 comparisons were evaluated.

## 3 Results

Results obtained from splitting the input dataset using our stratified sampling method over 2000 independent trials are shown in Table 1. The mean and standard deviation of each demographic category’s prevalence was calculated over all independent trials and compared to the prevalence in the input dataset and between the two subsets. For all demographic subcategories, the prevalence in the input dataset was matched in both the open and sequestered subsets within one standard deviation. For the category of image modality, the prevalence represents the percentage of patients with a given image modality available, and since many patients have images from multiple modalities, these percentages will not add to 100%.

**Fig. 2:**
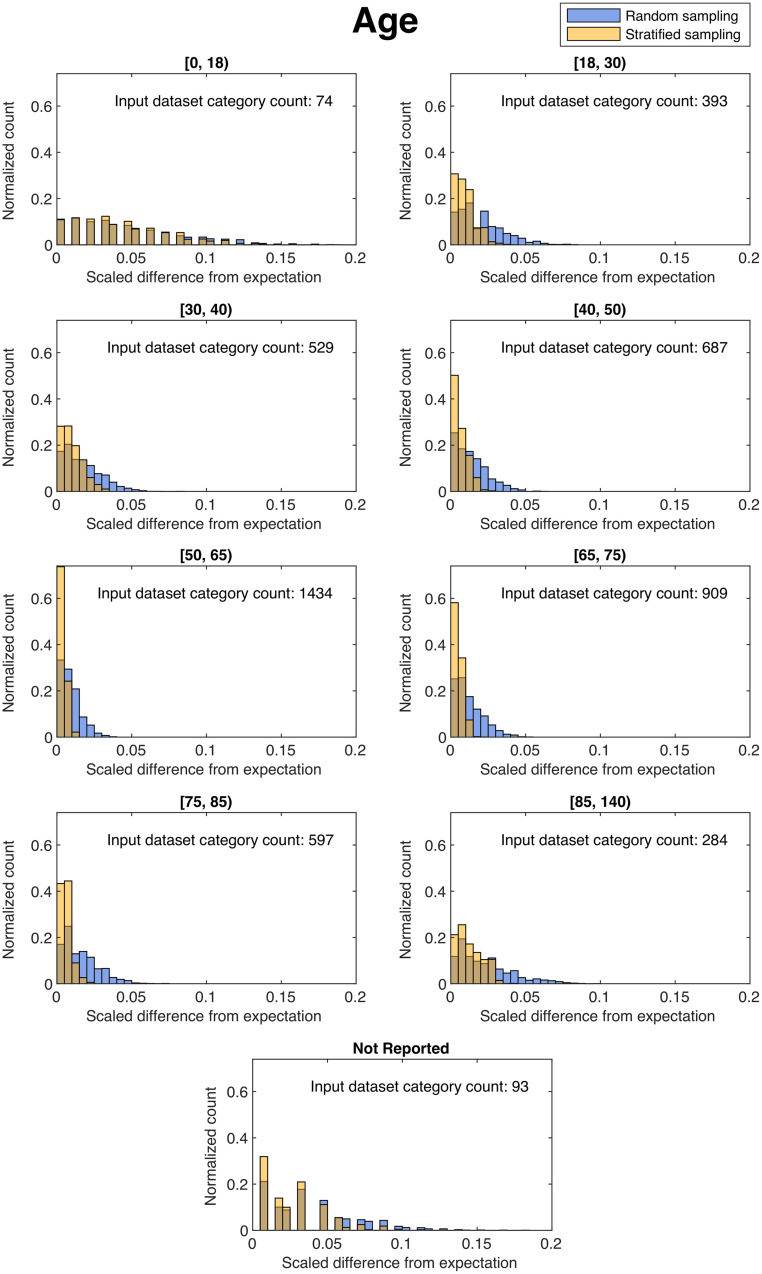
Histograms of the scaled difference from expectation for the category of age in the “Open” dataset after 2000 independent trials of dataset splitting using naïve random sampling (blue) and our stratified sampling method (yellow). Here, a narrower histogram indicates better balance, and a wider histogram indicates worse balance in a given subcategory over all independent trials.

**Fig. 3:**
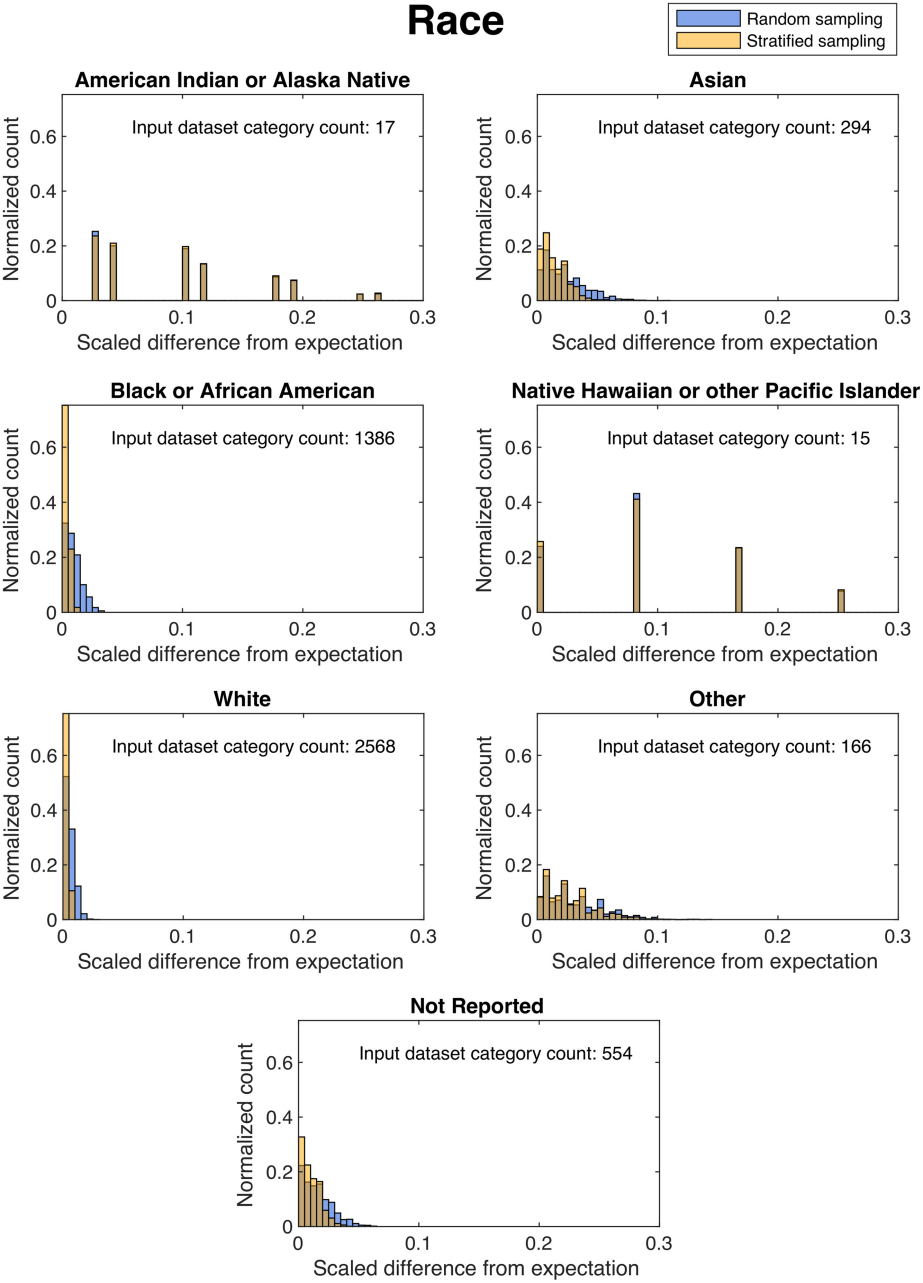
Histograms of the scaled difference from expectation for the category of race in the “Open” dataset after 2000 independent trials of dataset splitting using naïve random sampling (blue) and our stratified sampling method (yellow). Here, a narrower histogram indicates better balance, and a wider histogram indicates worse balance in a given subcategory over all independent trials.

Histograms of the scaled difference from expectation over 2000 independent trials for the categories of age and race in the “Open” subset (∼80%) are shown in Figs. 2 and 3. For most categories analyzed, sequestration by stratified sampling provided lower scaled differences from expectation, in general, than from the naïve randomization, as indicated by the narrower distributions for stratified sampling than naïve randomization. However, for some categories with low prevalence, such as race of American Indian or Native American, stratified sampling showed similar variation from expectation as the naïve randomization.

Results of the statistical comparison of the histograms of the scaled difference from expectation for all demographic subcategories categories in the “Open” subset using the one-tailed Mann–Whitney U test are shown in Table 2. Similar results were found for the “Sequestered” subset, despite having smaller relative sample size but are omitted for brevity. Statistical results support the qualitative summary that sequestration by stratified sampling provided lower differences from expectation, in general, than did the naïve randomization, with the exception of demographic subcategories with very low prevalence. P-values <0.05 were considered significant. Correction for multiple comparisons using Holm–Bonferroni did not change significance between the differences from expectation for any subcategory except the category of having an ethnicity of not Hispanic or Latino. As a result, for most demographic categories, our developed method of stratified sampling significantly outperformed naïve random sampling, providing more balanced demographic distributions on average.

**Table 2:** Results of the one-tailed Mann–Whitney U test, comparing distributions of the scaled difference from expectation in the “Open” dataset after 2000 independent trials of dataset splitting using naïve random sampling and using our stratified sampling method. P-values shown in bold were considered significant (p<0.05) and p-values with two asterisks were considered significant after Holm–Bonferroni multiple comparison correction. Here, statistically significant indicates the histogram of the scaled difference from expectation from stratified sampling algorithm was significantly narrower than the histogram from naïve random sampling.

Demographic subcategory	Input dataset count	Mann–Whitney U test result
**Age group**		
[0, 18)	74	p < 0.01**
[18, 30)	393	p < 0.01**
[30, 40)	529	p < 0.01**
[40, 50)	687	p < 0.01**
[50, 65)	1434	p < 0.01**
[65, 75)	909	p < 0.01**
[75, 85)	597	p < 0.01**
[85, 140)	284	p < 0.01**
Not reported	93	p < 0.01**
**Race**		
American Indian or Alaska Native	17	p = 0.70
Asian	294	p < 0.01**
Black or African American	1386	p < 0.01**
Native Hawaiian or other Pacific Islander	15	p = 0.29
White	2568	p < 0.01**
Not reported	554	p < 0.01**
Other	166	p < 0.01**
**Sex at birth**		
Female	2533	p < 0.01**
Male	2464	p < 0.01**
Other	0	N/A
Not reported	3	p = 0.52
**Ethnicity**		
Hispanic or Latino	499	p < 0.01**
Not Hispanic or Latino	4443	**p = 0.01**
Not reported	58	p = 0.57
**COVID-19 status**		
No	2602	p < 0.01**
Not reported	1	p = 0.29
Yes	2397	p < 0.01**
**Image modality**		
CR	2049	p < 0.01**
CT	910	p < 0.01**
DX	2596	p < 0.01**
MR	27	p < 0.01**

## 4 Discussion

We demonstrated, using our proposed method of multi-dimensional stratified sampling, that splitting an input dataset of 5000 COVID-19 patients into an 80% open dataset and a 20% sequestered dataset based on the variables of age, race, sex at birth, ethnicity, COVID-19 status, and image modality resulted in subsets that exhibited distributions very similar to those of the input dataset and each other. The high degree of similarity in the distributions indicates that the sequestration algorithm operated as expected. Moreover, the distributions of the differences from the expected values for the developed stratified sampling algorithm and naïve randomization indicated that the stratified sampling algorithm, in general, outperformed naïve randomization, providing more balanced distributions of demographics versus the demographic distributions obtained from naïve randomization.

It is important to note that while the analysis of the data focused on individual demographic categories, the developed method of multi-dimensional stratified sampling also controls the n-dimensional joint distribution of demographic categories. Thus, for each unique combination of demographic categories (e.g., 30 to 39 years old, Asian, Not Hispanic, COVID-19 positive, females, etc.), the stratified sampling algorithm separates that unique strata into “Open” or “Sequestered,” preserving the joint distributions. Additional work to evaluate the joint distribution balance of the entire MIDRC data commons is discussed in another publication.^27^

While the high degree of similarity in the distributions of variables across both subsets is promising, indicating that the proposed sampling method worked as intended, the ultimate goal in constructing a sequestered dataset for algorithm evaluation does not aim for perfect symmetry relative to the data going to the open dataset. Sequestration will provide an ongoing method to monitor and maintain a high level of similarity in the variable distributions, but perturbations in the demographics will also be purposely implemented to assure algorithm generalizability. Balanced distributions of demographic variables between open and sequestered commons allow for establishment and maintenance of a standard “MIDRC demographic” across both commons. This demographic profile is useful in evaluation of the bias and diversity of MIDRC data, which is discussed in another publication.^28^ Balance of the demographic distributions will also allow for users to sample similar data for training that can be later sampled for testing from the sequestered commons. However, all the data separated into each commons do not represent the subset on which training or testing will be directly performed. It instead represents an entire data commons that will need to be sampled by another method for selection of a cohort for a specific task. When an algorithm is tested using data from the sequestered dataset, test samples will be randomly drawn from the sequestered set according to the distributions related to the task (e.g., clinical question, clinical claim, and intended population), that is, the sequestered set in its entirety will not be used in the test. Furthermore, from the algorithm testing using sequestered data, only summary performance information will be reported back and not case-specific results. Methodology for this specific task-based sampling that is more directly related to performance of individual algorithms will be discussed in a future publication.^8^

The size of 5000 patients for the example set was selected to be approximately representative of a “typical” incoming data batch internal to MIDRC that would be proportionally split between the two commons. The 5000 patients were selected at random from the existing public data commons and are available for download on Ref. ^7^. Larger subsets of data would be expected to have relatively better achieved balance with stratification and smaller subsets would be expected to have relatively worse achieved balance with stratification, decreasing until it approaches naïve random sampling. However, this is shown within the current dataset within the smaller and larger strata investigated.

Assessment of machine learning algorithm performance is often achieved through methods such as k-fold cross validation or bootstrapping.^29^ These methods sample a limited dataset many times to test the algorithm on a variety of sample characteristics. More commonly, sampling for these methods is performed using naïve randomization. As such, naïve random sampling was an intuitive choice for a standard of comparison with the developed method of multi-step stratified sampling. Stratified randomization is an existing process used in separating training and testing datasets, but generally only allows for stratification across a single variable. However, balancing of multiple variables across public and sequestered datasets, from which cases cannot be made known, or used and replaced, is a task not typically considered in machine intelligence applications. Using the presented process, which sequentially steps through each branch until a single multi-variable stratum is obtained, balance across all possible combinations of the selected variables can be controlled. Similar processes are used in the construction of case and control populations in clinical trials, but these processes are typically conducted once, after collection of the entire population. Our process is implemented on each incoming MIDRC data batch, which are received on an ongoing basis. To the knowledge of the authors, this is the first application of multi-step stratified sampling to generate continually growing machine intelligence datasets.^15^^,^^19^^,^^20^

Stratification across a large number of variables also necessitates a relatively large initial dataset to be split to achieve its intended effects, i.e., achieving better balance than naïve randomization. This is evidenced in this work in the relatively lower difference in performance between random guessing and stratified sampling in subgroups of low prevalence. Once a subgroup population is too low to split 80:20 evenly, the process of assigning a patient to a specified subgroup is random with probability of 80:20. This is a known limitation for clinical trial stratification schemes.^20^ Having large data batches, on the order of thousands of patients, enables the stratification across multiple variables. Oakden-Rayner et al. provide an example of why stratification across many variables may be a beneficial approach since fewer variables are “hidden” and balance across many subgroups is monitored within MIDRC.^17^ The developed sequestration algorithm will provide an open-source tool for avoiding hidden stratification, through the use of a type of variable population or schema completion. Other, more sophisticated methods of sampling, such as minimization, could also be used for comparison to stratified sampling and may indeed outperform stratified sampling in certain circumstances, particularly when the number of patients relative to the number of strata is low. However, using a method of multi-step stratified sampling is intuitive for users to understand and may be simply modified to accommodate new stratification variables, increasing the general utility of an open-source tool.

With the explosion of machine intelligence algorithm development, validation of algorithm performance and generalizability has become increasingly important. As a result, many academic journals now encourage authors to make datasets and algorithms public when publishing. The creation of a large, common sequestered dataset that can be sampled for specific task-based algorithm performance evaluations could provide a new gold standard in the field of machine intelligence. Further, a sequestered database for algorithm testing could allow for expedited clinical implementation of algorithms developed for medical decision making if accepted by regulating bodies. This work outlines the process by which such a database has been developed for use in a multi-institutional data commons.

It is also important to discuss a few limitations of this study. The ability of stratified sampling to achieve a much higher degree of balance than simple randomization is highly dependent on the incoming dataset size and prevalence of a given demographic subcategory. Subgroups that are very rare in incoming data batches are likely to be rare across the entire data commons, and the ability to sample rare cases will always be limited by their availability in each data commons. Clinical trials that use similar stratification methods also note this be to be a limitation.^20^ While the currently developed algorithm samples based on the demographic categories of race, age, ethnicity, sex at birth, COVID-19 status, image modality, and clinical site, the algorithm will be adapted in the future to additional needs, including body site or patient location information. The authors also acknowledge that specific labels of race, ethnicity, or sex at birth may not adequately describe all populations or provide a clear correlate to genetic ancestry, which emphasizes the importance of oversight and monitoring of AI algorithms for equity in their applications to healthcare. As additional information and understanding of case descriptors are acquired, the sequestration algorithm will be modified to accommodate. In addition, while ensuring similar distributions of demographic characteristics may help to reduce potential algorithm bias, certain biases will still persist and must be acknowledged.

## 5 Conclusion

The creation of a sequestered dataset within MIDRC will allow for algorithm performance evaluation using large, verifiable multi-institutional data. This work outlines the process by which the sequestered dataset will be sampled from incoming data batches to ensure balanced variables in both the public and sequestered data. Using an example input dataset, both subsets were found to match the distribution of variables in the original data with a high degree of similarity.

## Data Availability

The software used in this paper, including MATLAB and Python translation of the algorithm, are publicly available on https://github.com/MIDRC/Stratified_Sampling/. The data used for this paper are maintained publicly at data.MIDRC.org. Additional information on the selection of the 5000 patients used in this study will be available on GitHub.
